# Comparison of US Panel Vendors for Online Surveys

**DOI:** 10.2196/jmir.2903

**Published:** 2013-11-29

**Authors:** Benjamin M Craig, Ron D Hays, A Simon Pickard, David Cella, Dennis A Revicki, Bryce B Reeve

**Affiliations:** ^1^Moffitt Cancer CenterTampa, FLUnited States; ^2^University of South FloridaTampa, FLUnited States; ^3^University of California, Los AngelesLos Angeles, CAUnited States; ^4^RANDSanta Monica, CAUnited States; ^5^Department of Pharmacy Systems, Outcomes, and PolicyCollege of Pharmacy, University of Illinois at ChicagoChicago, ILUnited States; ^6^Northwestern UniversityChicago, ILUnited States; ^7^Outcomes Research, EvideraBethesda, MDUnited States; ^8^University of North Carolina at Chapel HillChapel Hill, NCUnited States

**Keywords:** survey methods, community surveys, sampling bias, selection bias, Internet, data sources

## Abstract

**Background:**

Despite the increasing use of panel surveys, little is known about the differences in data quality across panels.

**Objective:**

The aim of this study was to characterize panel survey companies and their respondents based on (1) the timeliness of response by panelists, (2) the reliability of the demographic information they self-report, and (3) the generalizability of the characteristics of panelists to the US general population. A secondary objective was to highlight several issues to consider when selecting a panel vendor.

**Methods:**

We recruited a sample of US adults from 7 panel vendors using identical quotas and online surveys. All vendors met prespecified inclusion criteria. Panels were compared on the basis of how long the respondents took to complete the survey from time of initial invitation. To validate respondent identity, this study examined the proportion of consented respondents who failed to meet the technical criteria, failed to complete the screener questions, and provided discordant responses. Finally, characteristics of the respondents were compared to US census data and to the characteristics of other panels.

**Results:**

Across the 7 panel vendors, 2% to 9% of panelists responded within 2 days of invitation; however, approximately 20% of the respondents failed the screener, largely because of the discordance between self-reported birth date and the birth date in panel entry data. Although geographic characteristics largely agreed with US Census estimates, each sample underrepresented adults who did not graduate from high school and/or had annual incomes less than US $15,000. Except for 1 vendor, panel vendor samples overlapped one another by approximately 20% (ie, 1 in 5 respondents participated through 2 or more panel vendors).

**Conclusions:**

The results of this head-to-head comparison provide potential benchmarks in panel quality. The issues to consider when selecting panel vendors include responsiveness, failure to maintain sociodemographic diversity and validated data, and potential overlap between panels.

## Introduction

The dramatic growth of the use of panel vendors for online survey research has been described as “one of the most compelling stories of the last decade” [1]. A panel vendor is an organization that recruits and matches participants to a specified target audience of a survey to collect reliable quantitative information about the participants’ preferences and behaviors. They provide a wide range of services that allow researchers to expeditiously accrue survey respondents while protecting their anonymity, including maintaining the panel (recruitment and database), verifying identities (quality control), selecting and inviting panelists, compensating respondents for participation, and delivering panel entry and survey data for all invited panelists. In the field of survey research, the use of a panel vendor is attractive because of the widespread availability of Internet-linked devices (eg, tablets, smartphones, and laptops), enabling panel vendors to target difficult-to-reach populations. Online surveys through panel vendors allow researchers to collect data efficiently and inexpensively (eg, less than US $10/completed survey), but this approach has its shortcomings. A preliminary literature search revealed multiple studies comparing survey mode (eg, online, face-to-face, telephone, postal) [2-10]; however, systematic comparisons of panel vendors for online surveys were not found, which is particularly troubling because of their almost exclusive use of nonprobability recruitment methods.

Because most panel vendors rely on nonprobability-based recruitment, their samples have unknown representativeness of target populations. In an attempt to address this uncertainty, panel vendor users typically specify a target number of respondents along with selected demographic characteristics (quota sampling) and rely on poststratification adjustments (analytic weights) to compensate for nonresponse and noncoverage. That is, panel respondents are weighted so that the marginal distributions of gender, age, race/ethnicity, education, and other demographic characteristics match a target distribution (eg, US Census). Quota sampling is necessary because of concerns that standing panels differ from the general population in that they are more educated, have a higher income, and are more likely to be younger, white, and non-Hispanic [1].

Panel vendors often advertise that they have large and nationally representative panels for timely and accurate online surveys of the United States. In addition to favoring nationally representative samples, most survey researchers prefer that invited panelists respond quickly and accurately. This study conducted a head-to-head comparison of 7 US panel vendors by giving each the same task and comparing them along 3 basic quality criteria:

1. Data efficiency: Do their invited panelists respond quickly?
2. Data validity: Are their respondents who they say they are?
3. Panel representativeness: Do their respondents have similar characteristics to the US general population?

This paper excludes potentially relevant details beyond the survey screener (eg, dropout rates and survey completion), which may be examined in future research and will likely vary by survey instrument. The purpose of this study was to typify panel vendors and their respondents and to provide helpful information, while acting in accordance to contract restrictions. Therefore, panel vendor names were withheld to protect their anonymity and were assigned a rank-based identifier based on the proportion of invited respondents who consented (panel vendor [PV]; PV1 to PV7). All study procedures were approved by the University of South Florida Institutional Review Board (IRB # Pro00000076).

## Methods

### Panel Vendors

A standardized scope of work was described in the request for quotes (RFQ), including the following 7 requirements:

1. 1000 US adult respondents at less than US $10 per completed survey;
2. 18 demographic quotas filled using their own panel (no partners or brokers);
3. Third-party survey hosting with email invitation only (no river sampling, routing, or banners);
4. Panel entry data on each invited panelist (ie, date of birth, gender, race/ethnicity, socioeconomic status, health status, and geographic location);
5. Date and time of invitation and panel enrollment for all invited panelists;
6. Vendor affiliation with either the European Society for Opinion and Market Research (ESOMAR), the Council of American Survey Research Organizations (CASRO), and/or the American Association for Public Opinion Research (AAPOR); and
7. Vendor responses to the ESOMAR 26, an industry standard and a survey instrument designed to help research buyers of online samples [11].

All possible panel vendors were contacted and those who met the requirements were included. Some (if not most) panel vendors are panel or list “brokers” (ie, they outsource surveys to partners who then administer it to their own panels) and did not meet the requirements of this study. Requirements specified that all respondents be recruited from a single proprietary source of standing panelists (no partners) and be invited using a generic email invitation. Sampling from multiple panels or without invitation would have complicated the delivery of panel entry data on all invitees (requirement #4).

First, to identify all possible panel vendors, the third-party survey host (ie, website provider) was asked to recommend panel vendors based on their prior experience. Second, a review was performed of all panel vendor advertisements and membership to standard-setting organizations in online research, such as AAPOR, ESOMAR, CASRO, and Quirk’s Marketing Research Review [11-14]. Third, referrals were solicited from experts in the field based on their experiences with various panel vendors. Each time a potential panel vendor was identified, their website was evaluated to ascertain whether they met the study requirements.

As of March 2012, 134 panel vendors were identified and reviewed [15]. After removing panel brokers and those who could not meet the 7 requirements, RFQs were sent to 23 panel vendors; however, only 12 panel vendors provided a quote that met the 7 requirements. Once the project’s scope of work was agreed upon by the panel vendor representative, a contract was sent to the panel vendor legal team for review and approval, with a turnaround time of as little as 1 week or as long as 5 months. Among the 12 panel vendors, 5 declined, largely because of disagreements between the panel vendor legal team and account representatives about the scope of work. The time from start to finish—RFQ to signing—ranged from 3 weeks to 6 months for the successful 7 panel vendor collaborations.

### Survey Design

#### Overview

At launch, panel vendors sent generic email invitations directly to their respective panelists in a series of waves. For this study, response time is defined by the number of days from the time of invitation to the time of consent (ie, do their invited panelists respond quickly?). This measure is analogous to response time in postal surveys, in which response is intrinsically linked to consent because nonconsenting respondents rarely return postal surveys. In this study, some respondents closed their browser on the consent page or stated nonconsent, but returned later to consent and proceed with the online survey (ie, response time).

After consenting, respondents answered questions about their current US state of residence, ZIP code, age, birth date, gender, race, and ethnicity, and were assessed for 4 technical requirements that enabled participation in the survey [15].

#### Complete Pass-Through Information

Upon clicking the invitation link, a respondent identification number was sent (ie, passed through) to the survey website to identify the panelist for payment purposes.

#### Complete Panel Entry Information

Each panel vendor was required to deliver the time and date of invitation for each invited panelist, which was linked to survey responses by using the respondent identification number.

#### Complete or Valid Geolocation

The Internet Protocol (IP) address was invalid if it was for a proxy server or if its geolocation was unknown or outside the United States. Respondents were required to be within the 50 US states or the District of Columbia for this study.

#### JavaScript Enabled

JavaScript was required for the survey software to function as designed.

To assess discordance, survey responses were compared with each other (ie, age and birth date; state and ZIP code), with the panel entry data (eg, gender), and with IP address data (eg, state). Discordance in race and ethnicity was not assessed because of differences in questions used by panel vendors at panel entry. Proof of valid identity was defined as responses that meet the technical requirements of the survey and were concordant (ie, are their respondents who they say they are?).

The last page of the screener asked respondents about their annual income and educational attainment. Panel vendors were required to fill 18 demographic quotas. Taking into account the demographic quotas, the validated respondents were assessed as to their representativeness of the US population in terms of income, education, and current US state of residence based on US Census data [16,17].

### Statistical Analysis

To assess response time, the proportion of invitees who responded on the same day, next day, and second day by panel vendor were estimated. Although some panel vendors provided invitation times, no panel vendor listed time zones that would allow hourly analysis. In violation of scope of work, PV7 did not provide invitation dates and was excluded from the response time analysis.

To validate respondent identity, the proportion of consented respondents who failed to meet the technical criteria, failed to complete the screener questions, and provided discordant responses were estimated. Discordance in self-reported responses was based on 4 indicators: (1) gender differed from panel entry data, (2) reported age differed from reported birth date, (3) birth month and year differed from panel entry data, and (4) ZIP code differed from current US state. The proportion of consented respondents with each discordant indicator is reported along with 2 further indicators: current US state disagreed with IP state, and birth date disagreed with panel entry data. These indicators were excluded from the definition of discordance because of panel-specific issues. Specifically, the bulk of IP state data were lost for PV2 because of an error in the Web-based survey software. In addition, PV4 reported that the day of birth in their panel entry data defaulted to the first of the month for a large portion of their panelists.

Before examining representativeness, respondents across panels were compared on self-reported birth date and ZIP code to assess the potential for overlap within and between panel vendors (ie, respondents completed the survey multiple times because they were panelists in more than 1 panel or were enrolled multiple times in the same panel). Under independence, the probability of finding a specific ZIP–birth combination from 1 panel in another panel is the product of 3 factors: (1) combined sample size of all other panels (N), (2) proportion of respondents in all panels with that birthdate (Sbirth), and (3) the proportion of respondents in all panels with that ZIP code (SZIP) This probability increases with sample size, the proportion with that birth date, and the proportion with that ZIP code (S=N × Sbirth × SZIP). Potential overlap between a panel and all other panels due to chance was estimated by the mean of these probabilities and compared to the actual overlap between panel vendors. This is a conservative estimate because birth dates may naturally cluster within ZIP codes (eg, universities, retirement communities).

To assess representativeness among the respondents who passed the screener, sampling weights were applied to 18 demographic quotas by panel vendor. Each quota was defined by age (18-34 years; 35-54 years; >55 years), gender (male; female), and race/ethnicity (Hispanic; black, non-Hispanic; white or other, non-Hispanic). The proportion of the sample in 6 education categories, 8 annual household income categories, and 9 US Census Bureau divisions were estimated. For comparison, these estimates were presented alongside national estimates from the 2010 American Community Survey (ACS) [18,19].

## Results

**Figure 1:**
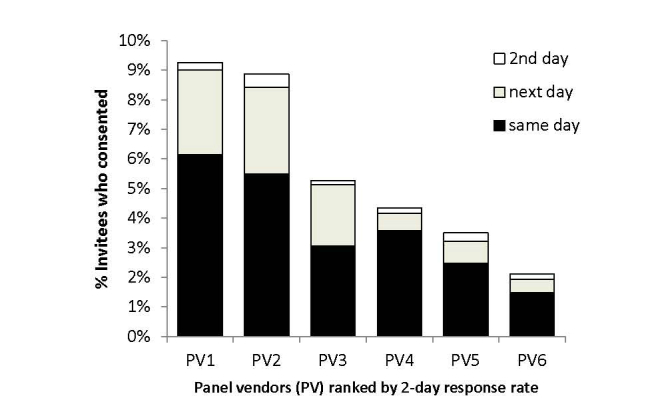
Proportion of invited panels who consented by day and panel vendor.

Figure 1 shows response rates by day among 6 of the 7 panel vendors, demonstrating that PV1 and PV2 had approximately double the response rates of the other panel vendors. The majority of invited panelists who consented did so on the day of invitation with further accrual on the next day, particularly when the invitations were sent in the evenings. Few consented on the second day after invitation.

**Figure 2:**
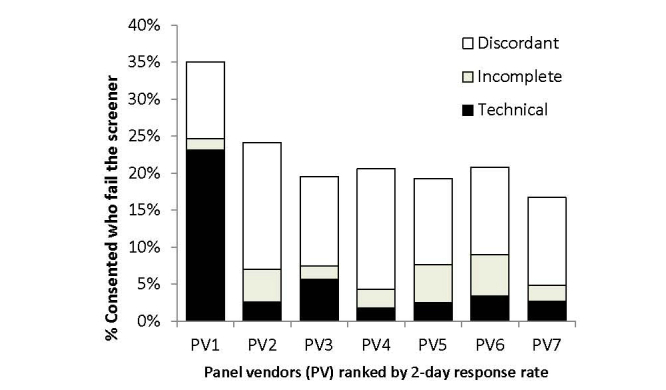
Proportion of consented respondents who failed the screener by reason and panel vendor.

Figure 2 describes the proportion of consented respondents who failed the screener. PV1 had a higher proportion largely because it was unable to find panel entry data for more than 1000 of those recruited, which was a technical requirement for this study. Only a few panelists failed to complete the screener after consent, which may be attributable to drop out or Internet disruptions. Aside from PV1’s panel entry data issue, approximately 20% of the consented respondents failed the screener, with the majority of loss because of discordant responses.

**Figure 3:**
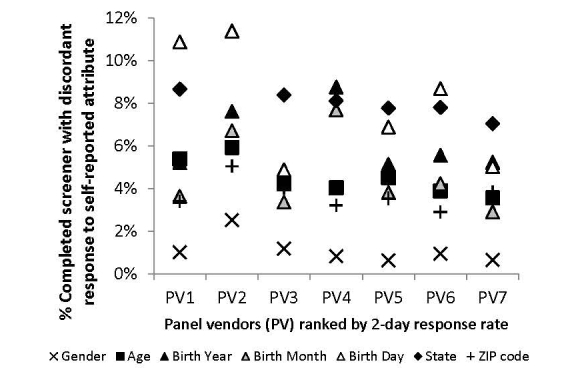
Proportion of discordant responses by self-reported attribute and panel vendor.

Figure 3 indicates the proportion of discordant responses among the respondents who completed the screener. Self-reported gender discordance ranged from 1% (PV5) to 2% (PV2), suggesting mostly agreement with panel vendor data. Self-reported age and ZIP code largely agreed with self-reported birth date and current US state with discordance ranging from 4% (PV7) to 6% (PV2) and from 3% (PV6) to 5% (PV2), respectively. Self-reported current US state using the IP state for 7% (PV7) to 9% (PV1) of respondents was unable to be verified, which was largely attributable to missing data. IP state was not captured for PV2 because of an error in the survey software.

The largest source of variability in discordance between panel vendors was birth date. Discordance in birth month and year for PV4 was twice that of PV3 (4% vs 9% and 3% vs 8%, respectively). Discordance in date of birth was greatest for PV1, PV2, and PV4. Because of 30% discordance in date of birth, PV4 acknowledged their use of the first of the month as their default, which largely invalidated the birth dates in their panel entry data. PV2 reported that some of their panelists intentionally report inaccurate date of birth to protect their identities.

To understand better the relationship among panel vendor samples, overlap of panel members was measured by the proportion of respondents in a panel vendor sample who reported a birth date and ZIP code identical to 1 or more respondents in another panel vendor sample. Although some repetition may be due to chance alone, Table 1 depicts systematic relationships between and within panel vendor–specific samples. Due to within-sample repetition, PV1 and PV6 may have allowed a small number of respondents to participate more than once (2% and 0.1%, respectively).

**Table 1:** Overlap^a^ within and between panel vendors (PV).

PV	PV1	PV2	PV3	PV4	PV5	PV6	PV7
Sample size	4183	3874	3158	2190	3644	2704	2154
PV1	1.7%	0.3%	6.2%	8.6%	8.1%	12.2%	5.6%
PV2	2.0%	0.0%	0.3%	0.5%	0.4%	0.6%	0.2%
PV3	6.3%	0.3%	0.0%	7.4%	4.8%	5.8%	3.2%
PV4	6.1%	0.3%	5.1%	0.0%	4.6%	4.7%	3.1%
PV5	8.6%	0.4%	5.5%	7.7%	0.0%	5.6%	4.2%
PV6	9.5%	0.3%	4.9%	5.7%	4.1%	0.1%	3.5%
PV7	4.5%	0.1%	2.2%	3.0%	2.5%	2.9%	0.0%
Any	23.2%	1.3%	18.6%	25.2%	19.7%	23.9%	14.6%

^a^ Overlap is measured by the proportion of screened respondents in the column PV sample who reported a birth date (ie, day, month, year) and 5-digit ZIP code identical to 1 or more screened respondents in the row PV sample.

Aside from PV2, all pairs of panel vendors had more than 2% overlap, suggesting recruitment from a common source. PV2 had the least overlap with any other panel vendor (1%). The greatest overlap was PV6 respondents who reported identically to 1 or more PV1 respondents (12%). Aside from PV2, the other panel vendors appeared to draw 15% to 25% of their sample from a common pool, likely because of panelists enrolling with multiple panel vendors. Assuming birth date and ZIP codes are unrelated, the predicted overlap because of chance ranged from 0.027% to 0.039%, which is less than the overlap observed between panels.

**Figure 4:**
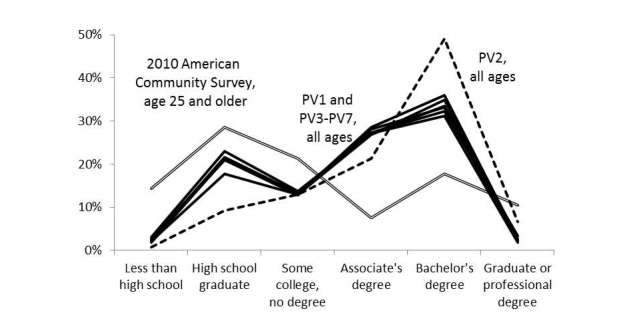
Weighted proportion of respondents who passed screener by educational attainment and panel vendor (PV).

**Figure 5:**
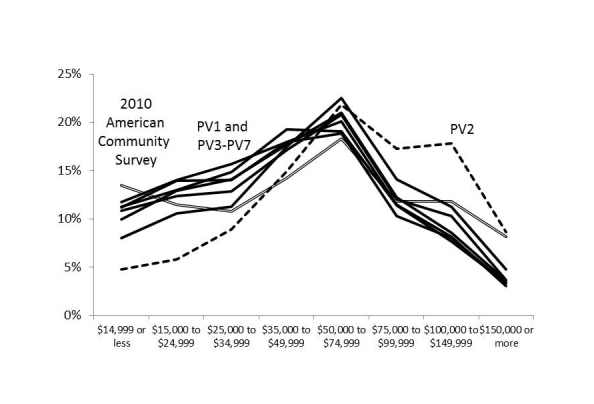
Weighted proportion of respondents who passed screener by annual household income in 2011 and panel vendor (PV).

**Figure 6:**
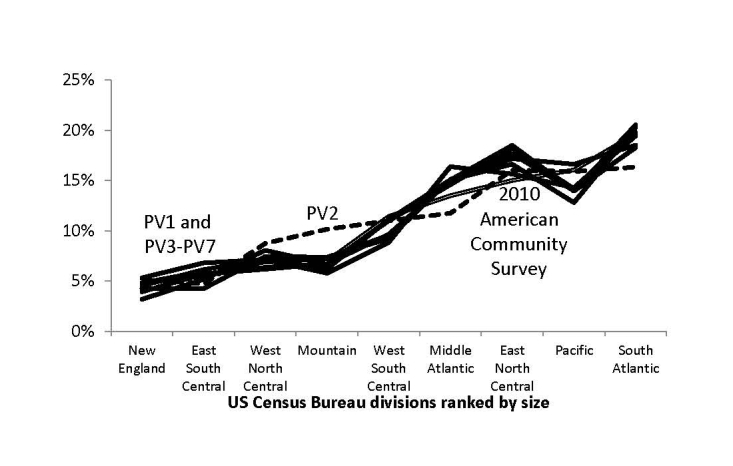
Weighted proportion of respondents who passed screener by US Census division and panel vendor (PV).

Figures 4-6 illustrate the representativeness of the panel vendor samples after applying demographic weights to the respondents who passed the screener. PV2 was distinct from the other 6 panel vendors (PV1 and PV3-PV7), favoring higher income and educational attainment as well as respondents in Western states. This relative skewness in PV2 improved representativeness in graduate education, incomes over US $150,000, and in the Pacific region, although sacrificing representativeness at high school education or less, incomes less than US $25,000, and along the Atlantic coast states.

Compared to the 2010 ACS estimates, the geographic differences seemed minor (all within a band of 4%). All panel vendors underrepresented adults who did not graduate from high school or had annual incomes less than US $15,000, with PV2 having the largest deficiency (–14% and –9%, respectively). However, 2010 ACS estimates included persons in institutionalized settings (eg, skilled nursing facilities, adult correctional facilities, and psychiatric hospitals) where Internet access may be restricted.

## Discussion

The increased use of panel vendors for online survey research makes it essential to understand the variability, differences, and shortcomings of panel vendors. The results of the study show variability in panel quality between the vendors and provide practical benchmarks for survey researchers. Points to consider when selecting panel vendors include responsiveness, failure to maintain sociodemographic diversity and validated data, and potential overlap between panels. Additionally, choosing to use an online survey panel offers advantages and disadvantages; therefore, the survey task itself should determine the mode used. In a matter of weeks and with a modest budget (<US $10 per complete), a general sample of US adults from 7 panel vendors was recruited. In addition to the 7 technical requirements for panel vendors, the project results may serve as benchmarks for panel vendor users who expect a 2-day 10% response rate with less than 10% of the consenting respondents failing the screener.

Discordance in birth dates is more of an issue compared with ZIP codes and other demographic characteristics. Six of the 7 panel vendors (PV1 and PV3-PV7) had a 25% overlap in respondents, which may explain why respondents in these 6 panel vendor samples provided similar educational attainment, geographic, and income responses. This study found that all panel vendor samples underrepresent low socioeconomic respondents, particularly PV2, which may motivate the greater use of socioeconomic status quotas in future work.

It has been argued that the representativeness of US panels is approximately that of random digit dialing surveys. In the 1970s, 89% of the US population had landline phones in contrast to less than 80% now [1]. Approximately 72% of the US population uses the Internet on a regular basis [1]. Even with recent increases in access to the Internet, online surveys have shown biases toward younger age, college education, higher socioeconomic status, English speaking, white, non-Hispanic ethnicity, literate, nonvisually impaired, and persons with low time costs [4,5,8,9,20,21]. A number of other studies have noted the nonrepresentativeness of panel entry data, perhaps attributable to self-selection or demographic and other unmeasured characteristic differences between panels and the general population [20,22-25]. These observable differences may affect the generalizability and self-selection for participation in panels may compromise external validity. For example, in the National Institutes of Health (NIH) Patient-Reported Outcomes Measurement Information System (PROMIS) project [26], despite a sample-matching methodology to ensure a sufficient representation in important subgroups, the resulting sample remained different from the 2010 US Census. Compared to the census data, the PROMIS unweighted general population sample was 5 years older, had a higher percentage of males, and had a higher percentage of those having a college education [21,27].

Most panels are recruited using nonprobability-based methods (eg, self-selection). Panel vendor users may apply sample-matching or quota-sampling approaches so that observable characteristics of respondents are similar to a target population. Despite potential similarity on measured variables, it is uncertain how these respondents compare to the desired population on unmeasured variables. For example, interest in participating in different types of surveys may further compromise generalizability. Interested participants may be different from uninterested participants in unobservable ways, as previous research has shown that women are more interested in health topics compared to men [28]. Finally, recruitment rates for participation in panels are only 33% or less [29], which may result in greater nonrepresentativeness of the potential panel-sampling frame. This limitation may be acceptable for research that is not intended to produce precise estimates of a target population.

Aside from panel attributes, response and completion rates vary between online and other modes of administration [6,20]. Advantages of online panels are similar to mailed surveys in that they can be completed at the convenience of the respondent, and the absence of an interviewer may reduce the possibility of social desirability bias. Disadvantages include the inability of respondents to obtain clarification for confusing questions or the inability of interviewers to know when a respondent does not understand the survey questions/tasks. Like postal surveys, respondents may complete the survey with minimal attention to the questions because of distractions and multitasking or they may not complete it at all. Unlike postal surveys, respondents may complete the same online survey multiple times, particularly if the study uses multiple panel vendors or a panel vendor with insufficient control. In addition, some respondents deliberately speed through online surveys just to obtain the incentive. For these reasons, it is important to consider the nature of the survey task before deciding whether an online survey is the best approach to a study.

There are many merits to population-based samples and modes for a variety of research questions and settings. Where population-based sampling is not attainable, a variety of weighting schemes can be applied to results of a panel survey in such a way as to minimize the bias introduced by lack of representation on key demographic characteristics. It is also noteworthy that some studies do not require representativeness to retain their internal validity. Experimental designs that test theory-driven hypotheses within a defined sample can legitimately test those hypotheses, even when the sample is not representative of a larger population (eg, oversampling). Similarly, testing the psychometric performance of questionnaires, particularly the item-level statistics associated with banks of questions that are used to define and measure an underlying concept, does not require demographic representativeness of the sample. Within this context, it is far more important that the sample be sufficiently heterogeneous in regards to covering the full range of the subject that is being measured. In other words, sample representativeness on socioeconomic variables is not always essential for good survey science. It depends on the nature of the study.

To our knowledge, this is the first study to perform a head-to-head comparison of panel vendors on 3 key criteria relevant to researchers: data efficiency, data validity, and panel representativeness. This study demonstrates the variability of panel quality and that no tested panel vendor performed well in meeting their claims of nationally representative samples and high data quality. In addition to the 3 basic criteria examined in this study, other criteria, such as cost, service, mode (eg, telephone), other countries, and technical capabilities, may be examined in future work. Online survey researchers can objectively apply these benchmarks to assess their own panel experiences and to improve the field of online survey research.

## Abbreviations

AAPOR: American Association for Public Opinion Research
CASRO: Council of American Survey Research Organizations
ESOMAR: European Society for Opinion and Market Research
IP: Internet Protocol
NIH: National Institutes of Health
PROMIS: Patient-Reported Outcomes Measurement Information System
PV: panel vendor
RFQ: request for quotes

## References

[1] Baker R, Blumberg S, Brick JM, Couper MP, Courtright M, Dennis M, Dillman D, Frankel MR, Garland P, Groves RM, Kennedy C, Krosnick J, Lee S, Lavrakas P, Link M, Piekarski L, Rao K, Rivers D, Thomas RK, Zahs D (2010). AAPOR Report on Online Panels.

[2] Berrens RP, Bohara AK, Jenkins-Smith H, Silva C, Weimer DL (2003). The advent of Internet surveys for political research: A comparison of telephone and Internet samples. Polit Anal.

[3] Duffy B, Smith K, Terhanian G, Bremer J (2005). Comparing data from online and face-to-face surveys. Int J Market Res.

[4] Grandjean BD, Taylor PA, Nelson NM (2009). Comparing an Internet panel survey to mail and phone surveys on “willingness to pay” for environmental quality: a national mode test. The American Association for Public Opinion Research (AAPOR) 64th Annual Conference, May.

[5] Hirsch O, Hauschild F, Schmidt MH, Baum E, Christiansen H (2013). Comparison of Web-based and paper-based administration of ADHD questionnaires for adults. J Med Internet Res.

[6] Leece P, Bhandari M, Sprague S, Swiontkowski MF, Schemitsch EH, Tornetta P, Devereaux PJ, Guyatt GH (2004). Internet versus mailed questionnaires: a randomized comparison (2). J Med Internet Res.

[7] Rao K, Kaminska O, McCutcheon AL (2010). Recruiting probability samples for a multi-mode research panel with Internet and mail components. Public Opinion Quarterly.

[8] Roster CA, Rogers RD, Albaurn G, Klein D (2004). A comparison of response characteristics from web and telephone surveys. Int J Market Res.

[9] Whitehead L (2011). Methodological issues in Internet-mediated research: a randomized comparison of internet versus mailed questionnaires. J Med Internet Res.

[10] Yeager DS, Krosnick JA, Chang L, Javitz HS, Levendusky MS, Simpser A, Wang R (2011). Comparing theaccuracy of RDD telephone surveys and Internet surveys conducted with probability and non-probability samples. Public Opinion Quarterly.

[11] ESOMAR (2008). 26 Questions To Help Research Buyers Of Online Samples.

[12] (2013). American Association For Public Opinion Research.

[13] (2011). Quirk's Marketing Research Review.

[14] (2013). Council of American Survey Research Organizations.

[15] Craig BM, Reeve BB (2012). Methods Report on the PROMIS Valuation Study: Year 1.

[16] United States Census Bureau United States Census 2010.

[17] (2012). Zip Code Tabulation Areas (ZCTAs).

[18] (2010). Selected Social Characteristics in the United States: 2010 American Community Survey 1-Year Estimates.

[19] (2010). Selected Social Characteristics in the United States: 2010 American Community Survey 1-Year Estimates.

[20] Eysenbach G, Wyatt J (2002). Using the Internet for surveys and health research. J Med Internet Res.

[21] Liu H, Cella D, Gershon R, Shen J, Morales LS, Riley W, Hays RD (2010). Representativeness of the patient-reported outcomes measurement information system Internet panel. J Clin Epidemiol.

[22] Brick JM (2011). The future of survey sampling. Public Opinion Quarterly.

[23] Sparrow N (2006). Developing reliable online polls. Int J Market Res.

[24] Sparrow N (2007). Quality issues in online research. J Advert Res.

[25] Wood FB, Benson D, LaCroix EM, Siegel ER, Fariss S (2005). Use of Internet audience measurement data to gauge market share for online health information services. J Med Internet Res.

[26] PROMIS Health Organization, PROMIS Cooperative Group PROMIS-29 Profile v1.0. Investigator Format 2008-2012.

[27] Sugiura H, Ohkusa Y, Akahane M, Sano T, Okabe N, Imamura T (2011). Development of a web-based survey for monitoring daily health and its application in an epidemiological survey. J Med Internet Res.

[28] Fox S, Rainie L (2000). The online health care revolution: How the Web helps Americans take better care of themselves.

[29] Callegaro M, DiSogra C (2009). Computing response metrics for online panels. Public Opinion Quarterly.

